# Double Fogarty balloon catheter technique for difficult to retrieve esophageal foreign bodies

**DOI:** 10.1186/s40463-018-0318-3

**Published:** 2018-11-20

**Authors:** Peng You, Sandra Katsiris, Julie E. Strychowsky

**Affiliations:** 1 Department of Otolaryngology-Head and Neck Surgery, Schulich School of Medicine and Dentistry, Western University, London, Canada; 2 Department of Anesthesia and Perioperative Medicine, Schulich School of Medicine and Dentistry, Western University, London, Canada

**Keywords:** Foreign body removal, Fogarty catheter, Balloon extraction, Rigid esophagoscopy, Pediatrics

## Abstract

**Background:**

Foreign body ingestion is common, especially in the pediatric population. Plans for retrieval should be tailored to the specific esophageal foreign bodies.

**Case presentation:**

We present a difficult to retrieve esophageal foreign body in a 3-year-old girl who ingested a 2 cm glass pebble. Intraoperatively, attempts using conventional optical forceps and retrieval baskets were unsuccessful due to the size and smooth texture of the object. A novel strategy using double Fogarty embolectomy balloon catheters for retrieval of blunt esophageal foreign bodies was devised and described.

**Conclusion:**

The double fogarty retrieval technique described appeared to be safe and efficacious, allowing for extraction of large esophageal foreign bodies under direct visualization.

## Introduction

Foreign body ingestion is common, with the majority of cases occurring in the pediatric population [1, 2]. Management of foreign body ingestions varies based on the shape and size of the object ingested, its location, and the patients’ age and size. Although the majority of pediatric cases involve accidental coin ingestion [1, 3], plans for retrieval have to be tailored to the specific foreign body ingested. An endoscopic approach with concurrent airway protection is favoured. On occasion, this may prove difficult due to the characteristics and size of the ingested item. Here, we report a case of foreign body retrieval with rigid endoscopy utilizing a novel double Fogarty balloon catheter approach.

## Material and methods

### Patient presentation

**Fig. 1:**
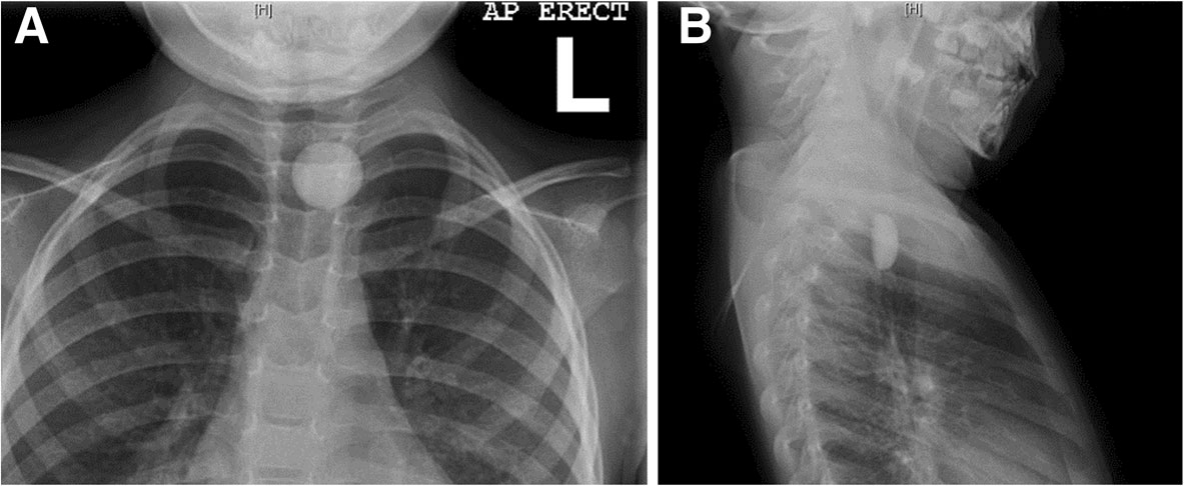
Chest X-Ray of a pediatric upper esophageal foreign body in anterior-posterior (**a**) and lateral (**b**) view. Letter “L” indicates the patient’s left side

A 3-year-old girl presented to the emergency department after ingesting a foreign body at daycare. The suspected object was a glass pebble. At the time of the incident, the patient did not have any aspiration symptoms and was not experiencing any dyspnea, vomiting, or hypersalivation. Her vital signs were all stable. A chest x-ray demonstrated the radiopaque foreign body in the proximal esophagus (Fig. 1). The patient was consented for rigid esophagoscopy and removal of foreign body under general anesthesia.

### Foreign body removal

**Fig. 2:**
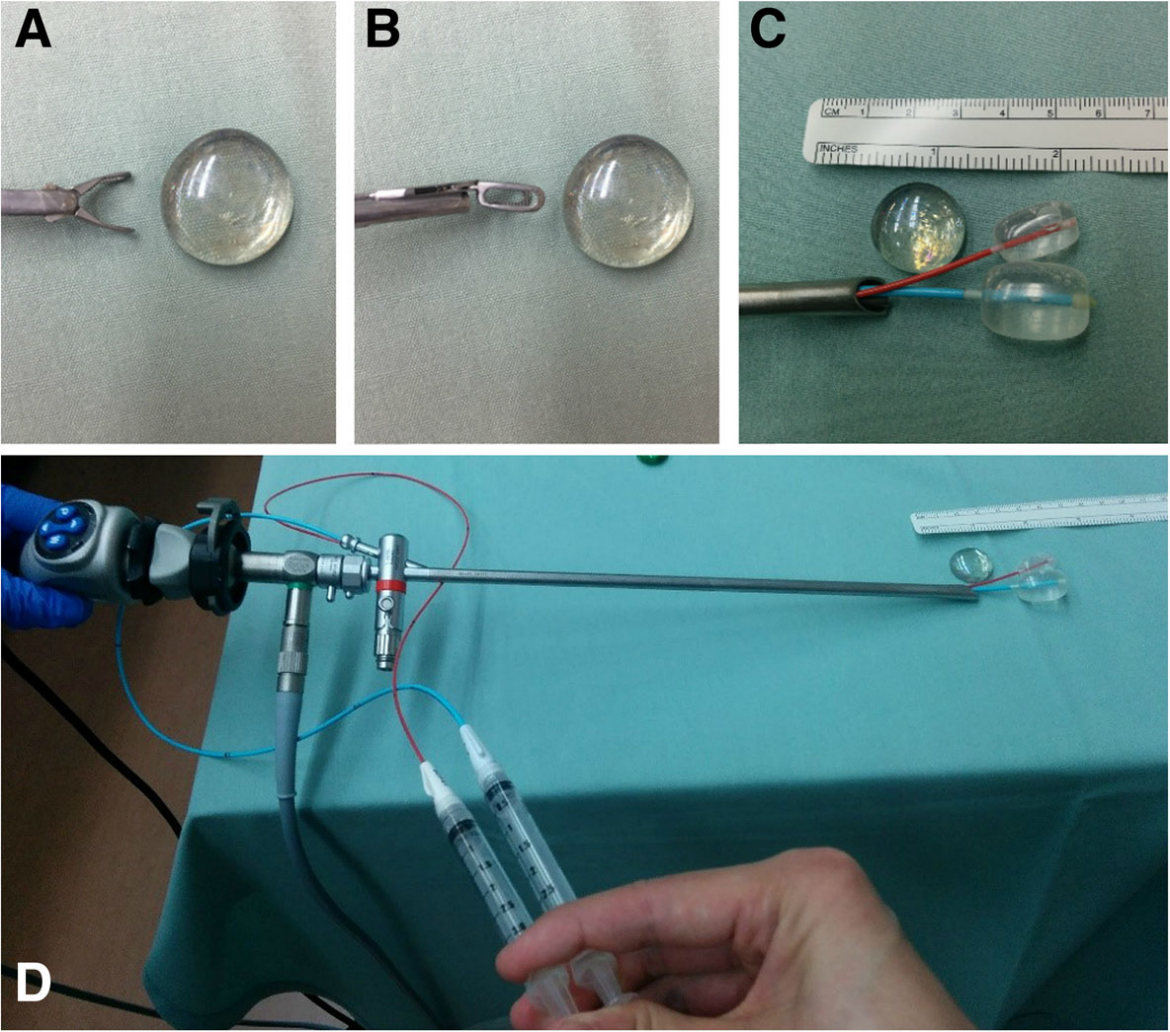
Blunt esophageal foreign body found to be too large for standard optical graspers (**a** and **b**). Retrieval was accomplished by using two Fogarty embolectomy balloon catheters threaded through the suction port of a pediatric rigid esophagoscope (**c** and **d**). The balloons were inflated distally and pulled back to remove the foreign body under visualization of the esophagoscope

The patient was taken to the operating room and intubated for airway protection. Thereafter, rigid esophagoscopy using a pediatric esophagoscope confirmed the presence of a semi-translucent smooth foreign body in the upper esophagus. Retrieval with the use of optical forceps was attempted, but the forceps were too small to grasp the glass pebble due to the pebble’s size and smooth surfaces. Removal using an urological retrieval basket was attempted, but it was also too small. Next, a size 6 French Fogarty embolectomy balloon catheter (13 mm diameter) was employed. With the endoscope in place, the catheter was threaded through the suction port of the rigid esophagoscope, passed distally to the foreign body, and maximally inflated. The balloon catheter proved to be ineffective since it would not engage the object, and instead slipped past the glass pebble due to the elasticity of the esophagus. At this point, a second Fogarty embolectomy balloon catheter (size 4 French; 9 mm diameter) was passed through the suction port alongside the size 6 French catheter (Fig. 2). Both balloon catheters were maximally inflated once passed distally to the foreign body. The catheters, along with the endoscope and esophagoscope, were then withdrawn from the esophagus under direct visualization. Moderate and steady traction allowed both balloons to engage and pull the foreign body along with them. The glass pebble was successfully pulled past the upper esophageal sphincter and promptly removed from the pharynx with McGill forceps. The esophagus was then inspected for residual foreign body or trauma.

## Results

Our technique for removal of a large glass pebble successfully extracted the foreign body with minimal trauma. There were no procedural complications. Following observation, the patient was discharged home in stable condition on postoperative day one.

## Discussion

Foreign body ingestion is commonly encountered, especially in children. In 2014, data from the American Association of Poison Control Centers documented close to 100,000 cases of foreign body ingestion by children and adolescents alone [4]. For pediatric cases, blunt objects such as coins are the most common foreign body ingested [1, 3]. The areas of impaction generally coincide with the locations of physiological narrowing. For the upper esophagus, this includes the upper esophageal sphincter, aortic arch, and left mainstem bronchus.

In the setting of blunt foreign body ingestion, impacted esophageal foreign objects should be removed within 24 h [2]. Delay in extraction decreases the likelihood of successful removal and increases the risk of complications such as perforation, with or without mediastinitis, retropharyngeal abscess and aortoesophageal fistula. In a single center case series, complication rates were found to be 14.1 times higher with foreign bodies impacted for more than 24 h [5]. Patients with complete esophageal obstruction with hypersalivation or inability to swallow liquids require more emergent intervention [1, 2].

Specific to blunt esophageal foreign bodies, non-endoscopic retrieval strategies have been proposed. This includes the use of Foley balloon catheters guided by fluoroscopy [6, 7] or esophageal bougienage [8]. However, drawbacks of non-endoscopic approaches include the risk of airway obstruction and esophageal injury due to lack of airway protection and direct visualization respectively. Therefore, it is generally recommended that foreign body extraction in children be performed under general anesthesia with endotracheal intubation to protect the airway [9–12].

For the present case, the smooth glass surface, as well as the size of the glass pebble, made retrieval with optical graspers impossible. A Foley catheter was too large to pass through the suction port of the rigid esophagoscope. Fortuitously, the profile of two Fogarty embolectomy balloon catheters was small enough to allow them to be threaded through the suction port of the rigid esophagoscope. We used Fogarty embolectomy balloon catheters of two different sizes since these were the largest catheters that would fit through the suction port together. Fogarty embolectomy balloon catheters have been used for airway foreign body extraction as well as in the setting of esophageal foreign bodies [13, 14]. The size and smooth surface of the foreign body necessitated the use of two Fogarty embolectomy balloon catheters concurrently, which has not been described in the literature. While the aforementioned case is unique, the described technique should be appropriate for other large blunt esophageal foreign bodies.

Alternative strategies in this scenario include proceeding with Foley balloon extraction with fluoroscopy guidance with the patient intubated or pushing the foreign body into the stomach. While the former requires considerable resources, the latter option too may require additional intervention from pediatric gastroenterology if the foreign body fails to pass through the rest of the alimentary tract. Instead, the double Fogarty approach allowed for removal of a large esophageal foreign body under direct visualization.

## Conclusion

The approach to extraction of pediatric foreign bodies must be tailored to the individual case. The double Fogarty embolectomy balloon catheter technique for retrieval of blunt esophageal foreign bodies appears to be safe and efficacious, allowing for extraction of a large foreign body under direct visualization.
